# Bioinformatics for the Human Microbiome Project

**DOI:** 10.1371/journal.pcbi.1002779

**Published:** 2012-11-29

**Authors:** Dirk Gevers, Mihai Pop, Patrick D. Schloss, Curtis Huttenhower

**Affiliations:** 1 The Broad Institute of MIT and Harvard, Cambridge, Massachusetts, United States of America; 2 Department of Computer Science and Center for Bioinformatics and Computational Biology, University of Maryland, College Park, Maryland, United States of America; 3 Department of Microbiology & Immunology, University of Michigan, Ann Arbor, Michigan, United States of America; 4 Department of Biostatistics, Harvard School of Public Health, Boston, Massachusetts, United States of America; University of California Davis, United States of America

Microbes inhabit virtually all sites of the human body, yet we know very little about the role they play in our health. In recent years, there has been increasing interest in studying human-associated microbial communities, particularly since microbial dysbioses have now been implicated in a number of human diseases [1]–[3]. Dysbiosis, the disruption of the normal microbial community structure, however, is impossible to define without first establishing what “normal microbial community structure” means within the healthy human microbiome. Recent advances in sequencing technologies have made it feasible to perform large-scale studies of microbial communities, providing the tools necessary to begin to address this question [4], [5]. This led to the implementation of the Human Microbiome Project (HMP) in 2007, an initiative funded by the National Institutes of Health Roadmap for Biomedical Research and constructed as a large, genome-scale community research project [6]. Any such project must plan for data analysis, computational methods development, and the public availability of tools and data; here, we provide an overview of the corresponding bioinformatics organization, history, and results from the HMP (Figure 1).

**Figure 1:**
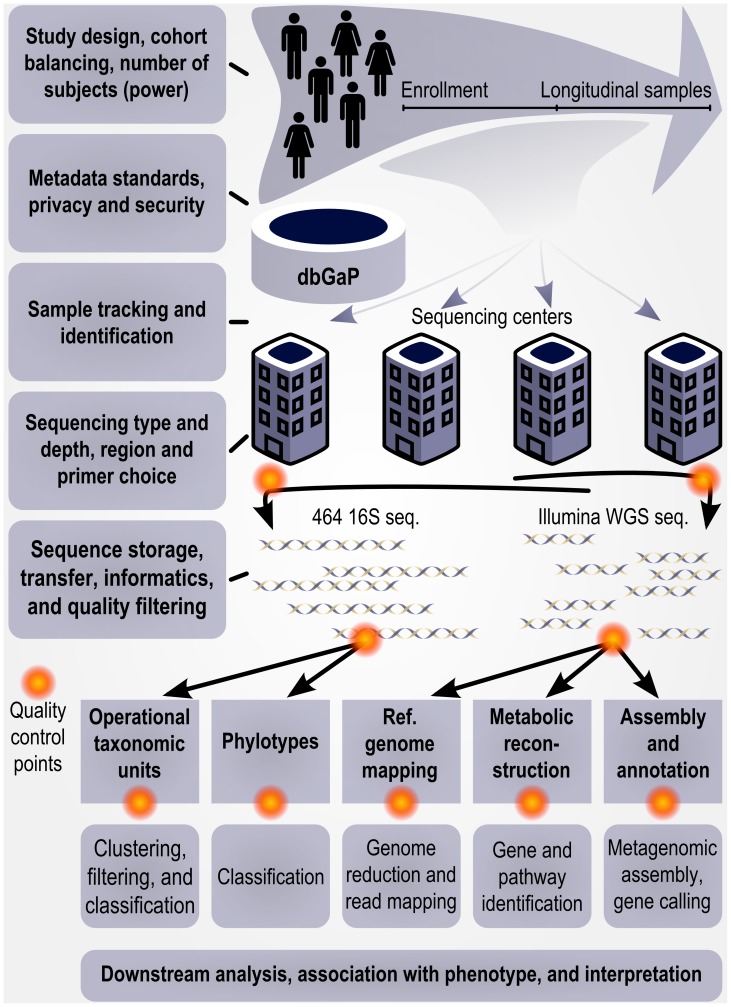
Bioinformatics in the HMP as a model for further studies of the human microbiome. Important computational considerations throughout the design, implementation, and analysis of a large human microbiome study such as the HMP; for details of the HMP's specific computational protocols, see [7], [42]. In the HMP, study design considerations included cohort balancing for gender and geographic location and recruitment of 300 individuals for adequate power. Subject metadata were protected and distributed through dbGaP [11], and up to three longitudinal samples were drawn from the microbiomes of 18 body habitats. These were tracked and sequenced at up to four distinct centers, including >5,000 16S rRNA gene datasets using 454 reads from the V1–3 and V3–5 hypervariable regions and >700 Illumina whole-genome shotgun datasets totaling over 8 Tbp of sequence. Quality control of sequences and datasets was performed at multiple points throughout data generation. Computational pipelines were developed and documented for each sequence data product as well as downstream analyses, with full results and protocols available at the HMP Data Analysis and Coordinating Center (http://hmpdacc.org).

One of the HMP's major goals was the generation of a baseline catalog of the microorganisms found in and on normal human hosts, which includes defining their normal patterns of phylogeny, taxonomy, biogeography, ecology, metabolism, and function. The HMP's study design included extensive sampling of the human microbiome from 300 subjects at five clinically relevant body areas (airways, skin, oral cavity, gastrointestinal tract, and vagina). Several specific body sites were sampled within each of these major areas, often at multiple time points, resulting in a total of 11,700 samples [7]. Advances in sequencing technologies over the course of the HMP allowed subsets of these samples to be explored both using marker gene sequencing [8] and through metagenomic shotgun sequencing of whole-community DNA [9], [10]. While these assays allowed the project's focus to scale from individual organisms to microbial communities as a whole, they presented daunting bioinformatic challenges. To date, the HMP has released over 100 million 16S rRNA gene reads and more than 8 Tbp of shotgun metagenomic sequences [7].

Before tackling the analysis of such a massive, heterogeneous sequencing data collection, early study design in the HMP planned for two critical and potentially conflicting bioinformatic considerations: subject privacy and rapid, public data release. Protection of human subjects for such a large cohort was handled by the EMMES Corporation, leveraging the resource of dbGaP [11] and emerging sequencing metadata standards [12] to provide quality control, security, and anonymous access to subject information for subsequent analyses. Deposition of nonprotected HMP data, its organization, and subsequently its public release were the mandate of the Data Analysis Coordination Center (DACC; http://hmpdacc.org), which was likewise formed early in the project. These steps were and are familiar aspects of genome sequencing and molecular epidemiology investigations, but once these data were protected and coordinated, the HMP was left with the task of developing appropriate and efficient analysis methodology.

The first bioinformatic challenges arose from the combination of large amounts of data with newly emerging sequencing technologies, particularly for 16S rRNA gene sequencing [13]. HMP data generation began in earnest during the spring of 2010, at which time the largest published microbiome datasets contained approximately 1–2 million 16S rRNA gene reads using the 454 platform [14], [15]. The HMP anticipated at least an order of magnitude more data, and these published datasets were themselves two orders of magnitude larger than previous studies. Identifying microbial membership and abundance using 16S rRNA gene sequencing has a long history [8], and many analysis tools and platforms were available [5], [16]–[18]. However, none were prepared to scale to the amount of data generated by the HMP. Major bioinformatic issues that were immediately apparent included high-throughput solutions for chimera detection in short reads 19, tackling increased sequence error rates [20], and adapting methods as the 454/Roche chemistry evolved [21], [22].

Computational analysis of shotgun metagenomic reads raised similar, even more extensive issues. The largest previous human-associated metagenomic data using the Illumina GA platform comprised some 0.5 Tbp [23], again several orders of magnitude more than commonly found in the literature at that time. Earlier work, in both environmental and human-associated communities [24]–[26] provided both critical biological insights and some analysis tools [27], [28], but while the former were vital for the HMP's later interpretation, the latter were not prepared for hundreds of samples comprising multiple terabases of 100 nt paired end reads from the Illumina GAIIx instrument. Over the course of the project, new analysis tools became available that partly addressed the challenges faced in this project: accelerated high-performance alternatives to BLAST [29], short read clustering [29], [30], and mapping approaches [31], [32], new interfaces to heterogeneous microbial community data [33], [34], and new de novo assembly software tailored to the Illumina data [35].

In order to address these challenges, as data generation began, the HMP specifically reached out to the bioinformatic community to create an analysis ecosystem around the anticipated large-scale datasets. The project aimed to bring together the extensive expertise and robust computational infrastructures of the large-scale sequencing centers with the many scientists actively developing new cutting-edge approaches for the analysis of metagenomic data. A Data Analysis Working Group (DAWG) was created, incorporating members of the four sequencing centers, the DACC, and researchers from the computational and microbiological research communities, many of whom volunteered their time out of enthusiasm for the project and its scientific potential. As the first HMP datasets became available in May of 2010, more than a hundred participants were organized into working groups focusing on different aspects of the data analysis process, including sequence quality control, assembly, annotation, metabolic reconstruction, and 16S-based studies. Through a series of conference calls, face-to-face meetings, computational breakthroughs, and hard work, the HMP DAWG developed and validated the series of bioinformatic solutions for human microbiome studies detailed below.

## A Comprehensive Human-Associated Microbial Census

Sequencing of the 16S rRNA gene is an effective method for interrogating the taxonomic composition of microbial communities. This gene is ubiquitous within the prokaryotic domain and can be effectively PCR-amplified from even previously unknown organisms. The analysis of microbial communities through the sequencing of 16S rRNA gene was common long before the influx of high-throughput sequencing (HTS) data [36], [37], making this gene one of the most highly represented within GenBank. HTS approaches to 16S rRNA sequence analysis typically include targeted Illumina or 454 reads of up to a few hundred nucleotides, each targeting uniquely identifiable variable regions of the gene that can be used as unique microbial identifiers [38]. The HMP planned to comprehensively characterize the taxonomic composition of the microbiome by averaging 5,000 454 FLX 16S rRNA gene sequences from all 300 subjects, 18 body sites, and multiple time points. This design, combined with more than a 1,000-fold increase in sequencing throughput over the course of the HMP, forced the consortium to develop novel tools for processing large 16S rRNA gene datasets, tackling issues specific to 454 sequence data quality, and addressing novel biological questions that were previously inaccessible due to limited sample sizes.

Approximately 6,000 samples for 16S rRNA gene sequencing by 454 FLX were collected at two clinical sampling centers, sequenced at four sequencing centers, tracked in combination with clinical and sample metadata, and the resulting data were finally deposited at the DACC, the short read archive, and dbGAP (http://hmpdacc.org/HMMCP and http://hmpdacc.org/HMQCP). Much of this data production was performed at a time where high-throughput 16S rRNA gene sequencing was relatively new and the quality of such data somewhat controversial [20], [39]. Since absolute certainty in individual base calls can be critical for microbial marker gene identification, the HMP developed a 16S rRNA gene sequence curation pipeline to reduce error rates while maintaining a large number of sequences of reasonable length. Both sample handling and sequence processing pipelines were optimized using benchmarks based on re-sequencing genes of known sequence. Several such “mock communities” were created including up to a few dozen organisms, assembled both from cells and from pre-quantified DNA, and comprising a wide range of microbial relative abundances. The resulting communities (BEI, Resources, Manassas, VA), sequencing protocols [40], and data (http://hmpdacc.org/HMMC) are now available, and together they provided a pipeline that reduced the sequencing errors from 0.6% to 0.02% and gave investigators greater confidence in the data [22].

Implementations of this pipeline are available in both mothur [16] and QIIME [41], HMP-funded software tools for microbial community data analysis. Both have undergone extensive revisions during the HMP to accommodate its data, incorporating robust software engineering strategies, improved algorithms, parallel processing, and efficient data storage. Both environments are constructed to be usable and to require minimal programming experience, and they provide rich analysis tools ranging from initial sequence handling to assessments of microbial ecology and sample metadata correlates. The HMP's deep and broad exploration of the human microbiome through 16S rRNA gene sequencing has thus already resulted in a number of biological insights [42], including the first comprehensive view of the normal pool of human-associated microbes (i.e., the “pan microbiome”). This has interesting ramifications for future studies, as one might ask what factors in a particular host select for different organisms from within the pan microbiome and may help to elucidate the mechanisms that result in specific assemblages of host-associated microbial communities.

An interesting question addressed by these data is the presence or absence of stable community configurations in different human body sites, such as enterotypes in the gut [43]. Identifying groups of highly similar microbial communities among many samples is a difficult unsupervised machine learning problem, akin to that of clustering or discovering molecular subtypes in cancer gene expression data [44]. Work to better understand the topic is ongoing, and the HMP's survey of many body sites offered the chance to contrast community organization within distinct ecologies. The vaginal microbiome, for example, has been observed to occupy one of five main states characterized by differing *Lactobacillus* spp. abundances [45]. This proved to be the case in the HMP as well [46], in contrast to a more complex continuum of community configurations occupied by the gut microbiota, particularly when meta-analyzed with the MetaHIT cohort [46], . As the presence of community types in distinct ecosystems may be influenced by environmental factors that can themselves vary continuously, such as diet [48], care must be taken in future computational efforts to reproducibly identify microbial community types within habitats where they do occur.

Taxonomic surveys through 16S rRNA gene sequencing are thus just a first step towards elucidating the role microorganisms play in our health and disease. We know that we are also colonized by archaea, micro-eukaryotes, and viruses, and further work is clearly needed to understand these “other” microbiomes and how they relate and interact with host-associated bacterial populations. In addition, taxonomy is only part of the story—the prevalence of horizontal gene transfer among microbes implies that an organism's function cannot be fully understood through taxonomy. The HMP thus began to address such issues by including a combination of culture-based studies and, for the first time, a tremendous resource of shotgun metagenomic data and analyses of the human microbiome.

## Putting the Pieces Together: Metagenomic Sequence Assembly

The taxonomic composition of the human microbiome is thus one step in understanding the role microbes play in our health, and it is well complemented by sequencing of microbial communities' entire genomic contents to catalog their biological functions. Thus, the HMP carried out extensive deep sequencing on a subset of its subjects and body sites using the Illumina platform (http://hmpdacc.org/HMASM). While portions of the HMP's 16S rRNA gene analysis were based on extensions of established experimental and computational approaches, this approach to whole-metagenome sequencing was a foray into new territory. The sequencing technology itself was (and still is) rapidly evolving, and metagenomic datasets of comparable size, read length, and ecological diversity did not previously exist. In the relatively short period between an initial pilot phase in 2007–2008 and the initiation of the production effort in 2009, Illumina read lengths increased by close to 30%, from 76 bp to over 100 bp. This also changed the error characteristics of the data being generated, which were already difficult to interpret in microbial communities containing hundreds or thousands of taxa. It thus necessitated development of a scalable end-to-end shotgun pre-processing and quality control pipeline, including duplicate read removal, quality and length trimming, host sequence removal, and whole-sample quality control. In the end, the HMP generated over 8 Tbp of raw sequence data, representing two lanes of paired-end Illumina sequencing for each of over 700 samples (targeting 10 Gbp/sample) as well as a small collection of samples, which were also sequenced with the Roche/454 instrument to investigate the impact of longer reads on metagenome assembly.

The design of this whole-metagenome sequencing experiment warrants a brief discussion. As the HMP was started, little information was available about the genomic diversity of the communities being assayed. The use of Illumina sequencing in metagenomics projects was still being debated, the main argument against this technology being the very short length of the reads being generated (just 100 bp compared to close to 400 bp achievable by Roche/454 and over 1,000 bp routinely achieved through Sanger sequencing). As detailed below, the feasibility of assembling the resulting data into large enough chunks to enable meaningful analyses was by no means obvious. At the same time, analyzing the reads themselves, rather than assembled contigs, was considered insufficiently accurate [49], although both assembly and read-based analyses ultimately proved successful. The choice of depth of sequencing, “just” two lanes of the instrument, was chosen to be sufficient to generate roughly 1-fold coverage of the *Escherichia coli* genome within gut microbiome samples (estimated to occur in most individuals at 0.1%–5% relative abundance [50]). The human distal gut was the body site for which the most prior knowledge was available due to extensive studies of the fecal microbiome, particularly due to insights from the MetaHIT project—a European-led study aimed at characterizing the human gut microbiome in health and disease [23].

Additionally, a major unknown factor regarding this shotgun sequencing was the level of human DNA “contamination” within whole-metagenome samples. With the exception of the distal gut, whose microbiome as estimated through fecal samples is almost entirely devoid of host cells, in other body sites it proved to be virtually impossible to sample the microbiome without also sampling host DNA. Even a minute level of host contamination can dramatically affect analysis of the associated microbiome, given that the DNA content of a single human cell is roughly a thousand-fold higher than that of a bacterial cell (a single human cell contains roughly 6 billion base-pairs of DNA as compared to just 4–6 million base-pairs found in a typical bacterial cell). As no experimental quantitative depletion protocols yet exist, in silico removal of human DNA was necessary not just to speed up the analysis but also to protect the privacy of the participants in the study. The resulting level of human contamination ranged from a low of <1% in stool to as high as >99% in some nasal and vaginal samples. Removal of these sequences (http://hmpdacc.org/tools_protocols/tools_protocols.php) and additional quality trimming reduced the total size of the HMP WGS dataset from 8.8 Tbp to 3.5 Tbp—less than half the data generated by the sequencing instruments, but approximately six times larger than the raw data of the MetaHIT project.

The HMP thus began exploring available bioinformatic options for metagenomic assembly during the generation of this massive dataset. The assembly of even isolated microbial genomes from Illumina data alone was (and still is) considered a difficult challenge, and the project was faced with the task of assembling a complex mixture of organisms present at widely varying levels of abundance. Genome assemblers are typically designed for the assembly of single genomes, expecting even coverage across a single large target contig, and they have only very recently begun to address the difficulty of handling metagenomic data [51]–[56]. Pilot HMP assemblies were thus highly fragmented, both due to polymorphisms between closely related organisms (e.g., mobile elements inserted in different genomic contexts) and due to abundant organisms being mistaken for genomic repeats.

To inform the development of the assembly strategy for the HMP, we performed a “bake-off” between the most commonly used assemblers at the time: SOAPdenovo [35], Newbler [57], ABySS [58], Celera Assembler [59], Velvet [60], and CLC (Cambridge, MA). The evaluation focused on both the contiguity of the resulting assemblies (number and size of contigs) and the accuracy of the reconstructed sequence, ascertained by alignment to genomes known to be present in our samples. Our efforts benefited from the availability of the “mock” metagenomic communities described above, but even so failed to identify a clear winner [7]—unsurprising in retrospect, as none of the assemblers we tested were designed for this task. It is important to note that both SOAPdenovo and Celera Assembler had metagenomics-specific features selectable through command-line parameters, however neither tool fully addressed all the challenges involved in the assembly of metagenomic data. Informed by these results, however, we proceeded to develop an assembly strategy around the SOAPdenovo assembler as used in the MetaHIT project in order to simplify comparisons to data generated in this earlier study.

With this protocol in hand (http://hmpdacc.org/doc/HMP_Assembly_SOP.pdf), the process of assembling the HMP's metagenomic samples progressed smoothly. The process was eventually run in parallel with data production itself, thanks to the distribution of computational effort between genome centers and community volunteers. The assembly of each of the ∼700 metagenomes required 4–6 h of computation time on large memory machines as well as the transfer to and from the DACC of large volumes of data. Some, although not all, of these processes proved to be automatable, but planning the hardware infrastructure both for distributed computing time and for very large-scale data transfers was a critical step in successful analysis.

The resulting assemblies proceeded both to downstream analyses such as gene identification and functional annotation and, as with all HMP datasets, to quality control [7]. Outlier samples whose assemblies differed significantly from others from the same body site in contiguity, number of ORFs, or level of human contamination were marked for exclusion from future global analyses. Singleton reads (those not included in any assembly) were pooled for assembly across multiple samples from each of the body sites. These body site-specific assemblies were targeted at the low abundance members of the community that were not sufficiently covered within a single sample. Surprisingly, identifying these unassembled reads was not a straightforward task, since the majority of modern assemblers do not report information about the placement of individual reads within assemblies, information that was instead reverse engineered by mapping original reads to assembled contigs using bowtie [32]. The resulting pooled assemblies provided substantial additional information, particularly in sparsely sampled body sites (those with high levels of human contamination) where only a small fraction of the reads could initially be assembled within individual samples.

As with the rest of the HMP's study design, development of this assembly strategy benefited from knowledge developed in earlier large-scale metagenomic projects, including the Global Ocean Survey [61], MetaHIT [23], and others. We were likewise able to determine which of the software tools from all available sources were suitable, if not yet ideal, for the assembly task in a metagenomic setting. Although additional tools were under development at the time or soon after, none were mature enough to support the production needs of the HMP. At the same time, the HMP made significant original contributions in the often overlooked and underappreciated engineering of robust, well-documented, and reproducible pipelines for processing and assembling metagenomic data. The protocols were tested and vetted by scientists from multiple independent institutions, both ensuring portability and enabling us to distribute the computational load among participants in the project. The resulting protocols, pipelines, and processed data are now available to any scientist to reproduce HMP results, adapt these methods to their project, or develop new algorithms using these data [7].

## Reading Between the Lines: Identifying Microbes, Genes, and Pathways

When this ultimately successful metagenomic assembly plan was first devised, it was not clear within the DAWG whether assembly would even be feasible for hundreds of metagenomes, each comprising short sequences from hundreds of different microbes. This raised the question of whether some analysis tasks could be carried out using only the unassembled short metagenomic reads. Read-based analyses, performed in parallel with the production assembly effort, in many cases generated results that were nearly the same as those obtained from the analysis of assembled contigs. Unassembled reads were used to assess which organisms were present in a community (http://hmpdacc.org/HMSCP), which genes (http://hmpdacc.org/HMGI), and which pathways (http://hmpdacc.org/HMMRC), complementing 16S rRNA gene-based taxonomic assessments and assembly-based gene annotations. Many genome-oriented analyses of interest were (and are still) impractical with short reads alone; synteny information is obviously unavailable, and methods requiring composition-based taxonomic assignment or discovery of novel organisms or gene families work best when provided with a longer genomic context [62], [63]. However, the perennial microbial community questions of “Who's there?” and “What are they doing?” both proved to be addressable through read-based analysis methods.

Determining the microbial composition of a community using unassembled short metagenomic reads has an increasingly long bioinformatic history [64]. Computational methods were and are increasingly successful at identifying the microbe(s) of origin for individual short reads [46], [65]. The HMP asked a new question, however, in assessing both the composition of the human microbiome and its genetic variation using read-based mapping to microbial reference genomes. After combining new HMP microbial isolates [66] with public databases, over 1,700 draft or finished microbial genomes were available to which the reads within each metagenome could be mapped [67]. Initial attempts at read alignment against this reference database revealed an immediate limitation of existing computational approaches: at the time this work was initiated, no evaluation of methods had been published for handling billions of reads targeting thousands of different genomes simultaneously, and a systematic assessment of speed and accuracy was first necessary. The HMP's resulting analysis indicated that since human-associated bacteria are phylogenetically well-covered by sequenced genomes [68], counting the number of reads mapped to each genome provided an accurate population census. This complemented results based on 16S rRNA gene sequencing to quantify community members, a task difficult to achieve precisely through 16S rRNA gene due to the varying copy number of the ribosomal operon in bacteria [69]. Also, unlike methods that directly classify each read into a taxonomic bin, these mapping results exposed single nucleotide polymorphism and structural variants within individuals' microbiomes [42]. This was a remarkable finding, whose ramifications remain to be explored: not only does every human genome harbor variants that can promote or prevent disease, every human microbiome might harbor personalized risk or protective microbial alleles as well.

Unassembled reads were also used to learn about microbial genes, pathways, and metabolic potential of the human microbiome [70]. A second DAWG subteam was formed focusing on metabolic reconstruction, which was tasked with functionally characterizing each read (whenever possible). Just as each read in a community metagenome arises from some particular organism, it in many cases also arises from an identifiable gene family. Thus, rather than using the strict nucleotide alignment of reads to the HMP genome catalog, the HMP investigated the use of translated BLAST (BLASTX) of individual reads against characterized protein families (e.g., KEGG [71] and MetaCyc [72]), whose annotated size exceeds that of available reference genomes). This approach to identifying the abundances of gene families in a community has been shown to be quite accurate [73]—but also led to computational challenges, since translated BLAST searches are much slower than the nucleotide mapping process used in reference alignment. The DAWG again undertook a systematic evaluation of accelerated translated BLAST technologies [70], several of which proved to be comparably accurate and sensitive for high-identity matches, and often thousands of times faster than a comprehensive BLASTX. Gene family abundances from the resulting catalog of alignments were reassociated into pathways for each metagenome, allowing the human microbiome to be described in terms of the metabolism being carried out by each community. This proved to be vital for understanding the ecological structure of the microbiome—the pathways carried by microbes within individuals' communities are far more consistent than the microbes carrying them, for example [42]. Some 50%–75% of short reads as yet remained uncharacterized, however, and functional databases must continue to expand to better capture the processes performed by microbes in situ in communities. A great deal of work thus remains to fully understand the metabolism and biomolecular functions of microbes, communities, and habitats throughout the human microbiome.

## The Road Behind, the Road Ahead

In addition to its scientific goals, a central mission of the HMP has been capacity building and resource sharing to enable further investigations of the human microbiome. The data resources of the HMP can continue to be mined as a baseline and contrast for targeted biological investigations, and they provide an extensive baseline for further computational tool development as well. Likewise, the documentation of both experimental and computational protocols throughout the project aims to guide future study designs for the human microbiome.

In particular, the HMP has emphasized the interpersonal variation of the healthy human microbiome, raising the bar for the breadth of human host populations and the number of microbial community samples that can be and should be investigated. As sequencing prices continue to drop and sample handling is automated, sampling levels comparable to those of the HMP may become possible even for individual research laboratories. Since the project has provided initial solutions to many of the accompanying logistical, technical, and informatic challenges, the next major computational hurdles will include development of appropriate analytical methodologies. Data visualization tools, machine learning, and modeling of longitudinal data will be critical to improving our understanding the human microbiome. One particular avenue of research that is critically needed is the development of statistical hypothesis testing methods that can incorporate nonnormally distributed, nonindependent data coupled with complex and diverse clinical histories, the absence of a core community across multiple individuals, and the extreme diversity of the typical host-associated microbiome [42].

16S rRNA gene-based studies currently provide the lowest cost means of assessing many microbial communities from large populations or longitudinal time courses [74]. Precisely defining microbial taxonomy and phylogeny from such studies has already been fraught with bioinformatic challenges in 454 reads of several hundred base pairs [22], and great care will be needed to accommodate sequencing errors and true biological ambiguity in shorter Illumina reads [75]. Primer design can critically influence the observability of different body sites' communities, both due to the universality (or lack thereof) of distinct regions' primers and their ability to differentiate human-associated portions of the microbial phylogeny [76]. Even if computational methods can optimize the choice of taxonomic marker genes, variable regions, primer design, noise and chimera reduction, binning, and clustering of operational taxonomic units, there remains the biological challenge of relating descriptors of microbial community structure to microbiome metabolism and function. Completion of microbial isolate genomes has accelerated along with microbial community sequencing, however [77], and a wealth of functional information remains to be tapped in their comparative genomics. Since the relationship between microbial phylogeny and function has been of interest for decades [78], this represents a rich area for exploration by computational methods.

Methods for metagenomic and metatranscriptomic sequence analysis, particularly by assembly, have likewise developed rapidly since the completion of the HMP. Assemblers capable of overcoming assumptions about genomic copy number [51], [79] and variation [54], as well as frameworks for the explicit study of metagenomic assembly (http://www.cbcb.umd.edu/software/metamos), have started to become available. Despite these developments, metagenomic assembly is far from being solved. Even in relatively low-complexity synthetic communities such as the 20-organism HMP “mock” systems 19, fewer than half of the genomes in the sample can be assembled with current software. Furthermore, assembly or annotation alone is not a sufficient end goal of most metagenomic projects, and new approaches need to be developed to allow both the extraction of biological information from the assembled data (e.g., identification of genomic variation, lateral gene transfer events) and the comparative analysis of assembled data across multiple communities. Finally, the generic term “assembly” encompasses many different use cases beyond the holistic assembly of entire metagenomes or transcriptomes. New approaches will need to be developed to address specific assembly tasks, such as targeted search and queries into metagenomic datasets, reconstruction of single genomes of interest [52] (e.g., identified by 16S rRNA or other genomic signatures), analysis of the population structure within a group of similar organisms (e.g., viral quasi-species), and relating metagenomes to functional data from transcriptomes or proteomes.

**Figure 2:**
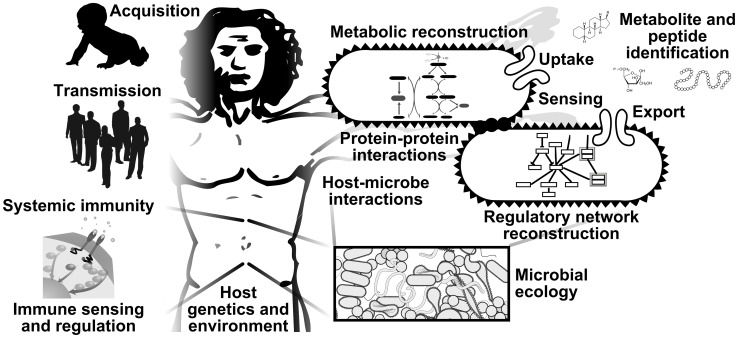
Topics in the study of the human microbiome with outstanding computational biology challenges. There remain many areas in the study of the human microbiome that will benefit from further bioinformatic efforts. At a whole-population level, the dynamics and stochasticity of microbiome acquisition at birth and its subsequent intersubject transmission must be characterized. As individual hosts, we each expose our microbiomes to unique genetic, dietary, pharmaceutical, and environmental perturbations, which in turn dictate systematic immune responses that are governed by individual sensing and regulatory biomolecular mechanisms. Within our microbiome, both host-microbe and microbe-microbe interactions dictate community ecology. These are governed by a variety of molecular mechanisms well-studied in model microbes including protein–protein interactions, metabolism, regulatory networks, and extracellular transport. In many of the most difficult assay types, such as whole-community proteomics or metabolomics, informatic challenges such as molecular identification remain to be overcome.

Integration of functional data from multiple complementary assays of the human microbiome, a process that has already begun in several studies [80]–[84], is thus one of the most exciting future challenges in microbial community bioinformatics (Figure 2). In order to translate our emerging understanding of the human microbiome into, for example, diagnostic or prognostic biomarkers, both broader pictures of the microbiome's epidemiology and deeper analysis of its biomolecular functions must be performed. A comprehensive study design might include an initial population survey generating thousands of 16S rRNA gene datasets, subsequent metagenomics, transcriptomics, proteomics, and metabolomics on a carefully selected subset of communities, and the combination of resulting data to identify which metabolites might be generated by transcriptionally and translationally active pathways in specific low- or high-abundance microbes. Longitudinal studies with carefully standardized clinical and environmental metadata [12] will likewise be critical for establishing the causality of microbial involvement in human disease and the microbiome's potential as a target for intervention or predicting response to treatment.

In the nearer term, just as the Human Genome Project introduced the need for scalable and sharable bioinformatic infrastructure, the HMP has reemphasized this need with its 100-fold greater sequence production. Repeatedly transferring such large datasets is at best inefficient and at worst impossible, and emerging cloud technologies represent a new opportunity to bring bioinformatics to the data rather than vice versa [85]. It is likely that the HMP data and computational tools will soon be available in one or more cloud environments, and this is a data analysis and delivery method that we encourage for future studies of the microbiome. Completion of the human genome has represented both a small step and a giant leap in bioinformatics and human health, and we hope that the HMP will represent a similarly solid foundation for future work.
